# *Oceanapia magna* Sponge Presents Dual Effect on the Gastrointestinal Motility of Rodents: *In Vitro* and *In Vivo* Assays

**DOI:** 10.3389/fphar.2020.572574

**Published:** 2020-12-23

**Authors:** Joedna Cavalcante Pereira, Indyra Alencar Duarte Figueiredo, Filipe Rodolfo Moreira Borges de Oliveira, Sarah Rebeca Dantas Ferreira, Giulyane Targino Aires Moreno, Tania Maria Sarmento da Silva, Ulisses dos Santos Pinheiro, Barbara Viviana de Oliveira Santos, Bagnólia Araújo da Silva, Fabiana de Andrade Cavalcante

**Affiliations:** ^1^ Programa de Pós-graduação em Produtos Naturais e Sintéticos Bioativos, Universidade Federal da Paraíba, João Pessoa, Brazil; ^2^ Curso de Farmácia, Universidade Federal da Paraíba, João Pessoa, Brazil; ^3^ Departamento de Química, Universidade Federal Rural de Pernambuco, Recife, Brazil; ^4^ Departamento de Zoologia, Universidade Federal de Pernambuco, Recife, Brazil; ^5^ Departmento de Ciências Farmacêuticas, Universidade Federal da Paraíba, João Pessoa, Brazil; ^6^ Departamento de Fisiologia e Patologia, Universidade Federal da Paraíba, João Pessoa, Brazil

**Keywords:** marine sponge, *Oceanapia magna*, spasmolytic, spasmogenic, intestinal transit

## Abstract

*Oceanapia magna* Santos-Neto, Nascimento, Cavalcanti and Pinheiro sponges are distributed across tropical worldwide seas. Some studies of marine products have shown interesting activities in smooth muscle models. Hence, we assessed the effect of the ethanolic extract of *Oceanapia magna*. (OC-EtOH) on acute toxicity and gastrointestinal motility (*in vitro* and *in vivo*) in rodent models. On guinea pig ileum, OC-EtOH induced a concentration dependent contraction on basal tonus, which was not inhibited by atropine, but in the presence of pyrilamine or verapamil, the effect was antagonized. Contrastingly, on KCl- or histamine-induced contractions, OC-EtOH presented a transient contraction followed by a concentration-dependent relaxation. Moreover, OC-EtOH presented a relaxant profile on cumulative curves to CaCl_2_ and tonic contraction induced by S-(-)-BayK8644, through Cav blockade. The acute toxicity assay showed that OC-EtOH (2,000 mg/kg, p.o.) did not present any sign of toxicity in female mice. Additionally, OC-EtOH presented antidiarrheal effect in mice, increased the intestinal normal transit and reduced the castor oil-induced intestinal transit. Thus, OC-EtOH presented a dual effect on guinea pig ileum promoting contraction through activation of H_1_ and Ca_V_, and relaxation through Ca_V_ blockade, besides the effect on upper gastrointestinal transit in mice, showing a potential medicinal use of this sponge in intestinal diseases such as diarrhea.

## Introduction

Natural products are considered a natural library of combinatorial chemistry that can provide substances with chemical and pharmacological diversity (Wang et al., 2011). The vast range of products available in the nature can be considered an important source of substances with potential therapeutic interest (Butler et al., 2014). Among the natural products diversity, several researchers are interested in studying natural marine products due to the varied production of compounds that may be used to treat various diseases (Movahhedin et al., 2014).

Marine natural products are sources of compounds that have demonstrated a plethora of biological activities, in both pre-clinical and clinical research, such as antifungal (D’auria et al., 2007), anticoagulant (Jung et al., 2007), antibacterial (Horie et al., 2008), anti-inflammatory (Chao et al., 2008), immunomodulatory (Yamada et al., 2007; Courtois et al., 2008), antiviral (Artan et al., 2008) and anticancer (Malyarenko et al., 2018; Rath et al., 2018). Additionally, spasmolytic activity has been described for the brown alga *Dictyota pulchella* (Queiroz et al., 2011), the green alga *Hydrodictyon reticulatum* (Gutierrez et al., 2012), and caulerpina, an alkaloid isolated from algae of *Caulerpa* genus (Cavalcante-Silva et al., 2013; Cavalcante-Silva et al., 2016).

*Oceanapia* genus (Oceanapiidae) presents about 100 sponges species distributed across the world’s tropical seas (Van Soest et al., 2015). Studies on *Oceanapia* genus are scarce in literature and only few biological activities have been described. *Oceanapia* species produce different classes of metabolites, including alkaloids, sphingolipids, steroids, acetylene, thiocyanate, among others (Ibrahim et al., 2013) that showed antifungal effect (Nicholas et al., 1999) and anticancer activity (Kijjoa et al., 2007).

*Oceanapia magna* Santos-Neto, Nascimento, Cavalcanti and Pinheiro is a recently described endemic species in Brazil. The word *magna* is an adjective derived from Latin and refers to the large size of this specimens (Santos-Neto et al., 2018).

There is considerable interest in investigating drugs obtained directly from natural products or their derivatives that act on the smooth muscle, once this type of muscle is present in the walls of various body organs, including blood vessels, stomach, bladder, airways and intestine. The regulation/deregulation of smooth muscle contractility plays a role in many pathophysiological processes such as hypertension, asthma, erectile dysfunction, renal and uterine cramping, diarrhea, and constipation (Webb, 2003).

Diarrhea is a process characterized by the increase of defecation frequency (more than 3 times in 24 h period), which may be watery or semi-liquid (World Health Organization, 2016). This disorder causes thousands of deaths per year, mainly in children under 5 years of age, which implies in a high cost for healthcare systems (Ndikubwimana and Ngendahimana, 2020). On the other hand, constipation is a condition that the individual presents less than three defecation episodes per week or evacuates hard, dry, and small stools, in a painful and difficult process to eliminate feces (Bielefeldt et al., 2016). This condition represents a significant economic burden. In the United States, the medical costs associated with constipation were estimated at United States $235 million in 2001, and about 95.3% of this costs were applied in the outpatient care (Nag et al., 2020). Therefore, there is an unexplored field that stimulates researchers to study natural products as tools to obtain new treatments and/or preventions to gastrointestinal diseases (Guo et al., 2014). Moreover, as described before, there is evidence that marine natural products present action on intestinal smooth muscle, what could represent an important contribution for human health, since marine products are known as a rich secondary metabolites source. Based on this, we decided to investigate a possible intestinal smooth muscle tone and/or antidiarrheal activities of the ethanolic extract from *Oceanapia magna* (OC-EtOH).

## Materials and Methods

### Product-Test

The ethanolic extract obtained from *Oceanapia magna* sponge (OC-EtOH) was used in this study. The sponge was collected by Petrobras (Petróleo of Brazil S/A), in May 2011, in Potiguar Basin/Rio Grande do Norte (04° 44.8945′ S/036° 25.4571′ W), with a 108 m depth, as part of Project: Inter-institutional Network Benthic Algae and INCT in Tropical Marine Environments - AmbTropic (CNPq No. 610013/2011-4) and identified by Professor PhD. Ulisses dos Santos Pinheiro, Federal University of Pernambuco, with the number of fall UFPEPOR 1551. After collected, the material was washed in running water and, then, separated. After, the sponge specimens were lyophilized and weighed. Subsequently, about 2 kg of lyophilized material was triturated and exhaustively extracted with ethanol and vacuum filtered with filter paper into a celite pad on Buchner funnel. The extraction solution was dried using a rotaevaporator to yield 10 g of material, providing a yield of 0.5%.

### HPLC Analysis

The *Oceanapia* extract was extracted in a solid phase extraction cartridge (SPE C-18), which was previously activated using 10 ml of methanol and 10 ml of ultrapure water. Approximately 128.0 mg of the extract was solubilized with 50 µL of MeOH and H_2_O (1:1) and added to the cartridge, washed with ultrapure water (10 ml). The retained material was eluted with methanol (10 ml) and the solvent was evaporated to obtain 13.9 mg of the methanolic fraction. This fraction was solubilized in MeOH (3.5 mg/ml) for analysis by HPLC-ELSD and quantification of β-sitosterol and stigmasterol.

Quantification of β-sitosterol and stigmasterol was performed using equipment from Shimadzu Prominence LC-20AT with an Evaporative Light Scattering detector–ELSD (Alltech Associates, United States), automatic injector SIL-20AC, oven CTO-20^a^ and degasser DGU-20A5. The chromatographic separation was performed with a column Luna C-18 (150 mm × 4.6 mm × 5 µm, Phenomenex). Methanol (HPLC grade) was used as mobile phase with flow of 1.0 ml/min, column temperature 40 °C, drift tube temperature of 70°C and a nitrogen flow at 2 ml/min. For filtering samples, 0.45 μm nylon filters were used (Whatman).

### Animals

For the experimental protocols, 168 Swiss mice (*Mus musculus*) weighing 31.8 ± 0.4 g and 63 guinea pigs (*Cavia porcellus*) weighing 370.5 ± 6.1 g, of both sexes, were used. All animals were housed at “Professor Thomas George” Bioterium of the Instituto de Pesquisa em Fármacos e Medicamentos (IPeFarM)/UFPB. The animals were kept in a 12-h light-dark cycle, with monitored temperature (21 ± 1°C), and unrestricted access to food and water. During *in vivo* experiments, researchers knew about the distribution of animals in the experimental groups. All the experimental procedures were formerly authorized by the Ethics Committee on Animal Use (CEUA)/UFPB, certificate no. 146/2015.

### Solutions and Drugs

Magnesium sulfate (MgSO_4_), potassium chloride (KCl), calcium chloride (CaCl_2_), glucose, sodium bicarbonate (NaHCO_3_), sodium chloride (NaCl), and sodium dihydrogen phosphate (NaH_2_PO_4_) were purchased from Vetec Química Fina Ltda. (Brazil). Atropine (99%), (S-(−)-Bay K8644), chloride verapamil, pyrilamine, carbamylcholine hydrochloride (CCh), Cremophor®, and histamine were obtained from Sigma-Aldrich (Brazil). Carboxymethylcellulose was obtained from Formula (Brazil). Castor oil was obtained from Farmax (Brazil). Loperamide (99%) was obtained from Janssen Cilag Farmacêutica Ltd. (Brazil) and activated charcoal was obtained from Proquímicos (Brazil). All substances were diluted in distilled water, and the extract was solubilized in Cremophor^®^ (3%), whose concentration never exceeded 0.01% (v/v). At this concentration, this chemical is devoid of contractile or relaxant effects on the studied organ, according to previous obtained data (data not shown).

### Measurement of Contractile Response

For isotonic contractions recording, segments of guinea pig ileum were suspended longitudinally by cotton yarn in organ baths (5 ml) and connected to an isotonic lever coupled to a smoked-drum kymograph (DTF, Brazil). Isometric transducers (TIM 05) coupled to an amplifier (AECAD04F) and connected to digital acquisition system AQCAD 2.1.6 from AVS Projetos (São Paulo, SP, Brazil) were used to record isometric contractions. To verify if the spasmolytic effect of OC-EtOH was reversible, the organ preparation was washed with the physiological solution and, after 30 min, a new contraction was induced in order to observe if organ responsiveness was altered (data not shown). In the cumulative concentration-response curves a new concentration of standard drugs or extract were only added when a plateau of the previous concentration was obtained.

### Pharmacological Experiments

#### Effect of OC-EtOH on Basal Tonus of Guinea Pig Ileum

The animals were fasted for 18 h before the euthanasia and subjected to decapitation using a guillotine. The abdomen was opened and an ileum segment of approximately 15 cm in length was removed and placed in a Petri dish containing a modified Krebs nutrient solution at 37°C, at a pH of 7.4, and gassed with carbogen (95% O_2_ and 5% CO_2_). Segments of this organ measuring approximately 2 to 3 cm were suspended in organ baths (5 ml). During the stabilization period (30 min) the organs were washed every 15 min by modified Krebs solution (mM): NaCl (117.0), KCl (4.7), MgSO_4_ (1.3), NaH_2_PO_4_ (1.2), CaCl_2_ (2.5), NaHCO_3_ (25.0) and glucose (11.0) at 37 °C and bubbled with a carbogen mixture in a resting tension of 1 g. After stabilization, an isotonic contraction with 40 mM KCl was induced to verify organ functionality. The preparations were washed just before adding OC-EtOH. Then, OC-EtOH was added and its effect on basal intestinal muscle tonus observed. A cumulative concentration-response curve to OC-EtOH was induced. The results were evaluated by contractile amplitude percentage response of OC-EtOH. The values of extract concentrations able to produce 50% of maximal effect (EC_50_) were calculated by non-linear regression.

#### Effect of OC-EtOH on KCl, Carbachol or Histamine-Induced Tonic Contractions

To evaluate the relaxing effect of the extract against different contracting agents, guinea pig ileum was obtained as described above. After stabilization period, 40 mM KCl, 10^–6^ M histamine or 10^–5^ M CCh was added to promote an isometric contraction in different preparations. To obtain the contraction plateau, the preparation continued in the presence of the contractile substances (approximately 10 min) then the organ was washed. After 30 min elapsed, a new contraction was performed with the same contractile agents as before to add OC-EtOH cumulatively (0.1–2,187 μg/ml). Relaxation was expressed as the reverse percentage of initial contraction elicited by the contractile agents. EC_50_ was obtained graphically from the concentration-response curves.

#### Evaluation of Muscarinic and Histaminergic Receptors and Ca_V_ Participation in Spasmogenic Effect of OC-EtOH

After the stabilization period, isotonic contractions were induced with 40 mM KCl to check the organ’s functionality and, after 15 min, two cumulative concentration-response curves of amplitudes similar to histamine, CCh (positive controls) or OC-EtOH were induced. Then, in the absence of the contractile agents or the extract, atropine (muscarinic antagonist, 10^–9^ and 3 × 10^–8^ M), pyrilamine (H_1_ receptor antagonist, 10^−9^–3 × 10^–7^ M) or verapamil (Ca_V_ blocker, 10^−7^–10^–6^ M) was incubated for 15 min, in independent experiments (Ghayur and Gilani, 2005). After this period, a new cumulative concentration-response curve for CCh, histamine or OC-EtOH was obtained in the presence of atropine, pyrilamine or verapamil, in order to evaluate the participation of the muscarinic and histaminergic receptors, as well as th Ca_V._

The results were evaluated by comparing the percentage of contractile response in presence and absence of antagonists and blocker. The inhibitory effect was evaluated based on the analysis of EC_50_ values and E_max_ of OC-EtOH, which were calculated from the concentration-response curves obtained as described above.

#### Effect of OC-EtOH on KCl-Induced Tonic Contraction in Presence of Pyrilamine

Guinea pig ileum was obtained as described. After the stabilization, the preparation was washed and, after 15 min, pyrilamine was pre-incubated for 30 min to observe if the spasmogenic component of extract relaxation was removed. Then, an isometric contraction was induced with 40 mM KCl and, during sustained tonic phase (8–10 min), OC-EtOH was added cumulatively (0.1–729 μg/ml). The relaxation produced by OC-EtOH was expressed as percentage contraction reverse produced by contractile agent. The EC_50_ values were calculated by nonlinear regression.

#### Effect of OC-EtOH on CaCl_2_-Induced Contractions in Despolarizing Medium Nominally Without Ca^2+^

After the stabilization period, a nominally Ca^2+^-free depolarization solution (with 70 mM KCl in equimolar exchange for NaCl) was used to swap the modified Krebs solution, for a period of 45 min, to obtain an isotonic contraction. Then, CaCl_2_ was added cumulatively to obtain a concentration-response curve, this step was performed in duplicate. Subsequently, OC-EtOH was pre-incubated at different concentrations and a new contraction curve was performed in the presence of CaCl_2_ (Van Rossum, 1963) to assess the involvement of Ca_V_ in the relaxing effect of the extract. The maximal contraction obtained with the first CaCl_2_ concentration-response curve was considered to be 100% (control), and all contractions were assessed in reference to it.

#### Effect of OC-EtOH on S-(-)-Bay K8644-Induced Tonic Contractions

To determine the Ca_V_ subtype involved in the extract relaxation activity, guinea pig ileum was obtained as described above. After stabilization period, pyrilamine was added for 30 min, to remove the spasmogenic component of the extract, then, 15 mM KCl was added for 10 min to promote partial depolarization of the ileum (Conte-Camerino et al., 1987). Subsequently, S-(-)-Bay K8644 3 × 10^–7^ M, a selective Ca_V_1 agonist, induced a isometric contraction. During tonic phase of contraction, verapamil (positive control) or OC-EtOH (0.1–729 μg/ml) were added cumulatively. The relaxation was expressed as percentage of initial contraction reversal produced by S-(-)-Bay K8644 and EC_50_ value was calculated by nonlinear regression.

#### Behavioral Pharmacological Screening and Evaluation of Acute Toxicity of OC-EtOH in Mice

For evaluation of acute toxicity, we followed the methodology described by Organization for Economic Co-operation and Development (OECD) no. 423/2001 (OECD, 2001). Initially, three female mice were used, which, after fasting for 4 h, were treated with OC-EtOH (single dose of 2,000 mg/kg, p.o.) or saline +0.9% Cremophor^®^ (control, p.o.). Animals were also evaluated for 14 days for survival analysis purposes, in order to estimate the extract dose that kills 50% of animals tested (LD_50_).

After administration of OC-EtOH, a series of behavioral parameters were observed during first 4 h, such as hyperactivity, aggression, tremor, convulsion, piloerection, sedation, ataxia, catatonia, analgesia, loss of corneal and headset reflex, dyspnoea, ambulation, scaling, abdominal contractions, bleeding, self-mutilation and nausea-like behaviour, such as pica (adapted from Almeida et al., 1999; Batra and Schrott 2011). The ponderal evolution was also measured before treatment, 7 and 14 days after treatment. This protocol was performed twice, in different experiments.

#### Effect of OC-EtOH on Upper Gastrointestinal Transit in Mice

After 12 h of food restriction, 0.9% NaCl plus Cremophor® (10 ml/kg, negative control), atropine (2 mg/kg, positive control) or OC-EtOH (31.25; 62.5; 125, 250, 500 and 1,000 mg/kg) were administrated orally in male and female mice separated into three groups (n = 6, each). After 30 min, 5% activated charcoal solubilized in carboxymethylcellulose 0.5% (0.01 ml/g) was administered orally. After 30 min of administration of the marker (activated charcoal), the animals were euthanized by decapitation using guillotine, and an incision was made in the animal’s abdomen to remove the small intestine and measure the distance traveled by the marker. The same methods were used as previously, but 30 min before the administration of the activated charcoal, the castor oil (0.01 ml/g) was added orally (Hsu, 1982; Than et al., 1989). The results were expressed as a percentage of the distance covered by the marker in relation to the total length of the small intestine. The inhibitory effect that was exerted by OC-EtOH was evaluated based on an ED_50_ analysis.

#### Effect of OC-EtOH on Castor Oil-Induced Diarrhea in Mice

Animals were treated orally with 0.9% NaCl plus Cremophor^®^ (10 ml/kg, negative control), loperamide (10 mg/kg, positive control) or OC-EtOH (62.5; 125, 250, 500 and 1,000 mg/kg) in different groups (n = 6, each). Then, to induce diarrhea, castor oil (0.01 ml/g) was administered by gavage after 30 min of the initial treatments. These same animals were placed in boxes lined with white paper, separately, and observed for 4 h uninterrupted. The number of stools were assessed, as well as their consistency, and classified as solid or liquid to determine the total number of stools and the total quantity of liquid stool episode (Awouters et al., 1978). The inhibitory effect of OC-EtOH was evaluated based on the ED_50_ value, which is the dose of a drug that produces 50% of its maximal effect.

### Statistical Analysis

The results were expressed as a percentage of the mean ± standard error of the mean (SEM) and statistically analyzed by the one-way ANOVA followed by Bonferroni’s post-test for multiple comparisons. The null hypothesis was discarded when *p* < 0.05. The EC_50_ and ED_50_ values were calculated by nonlinear regression. The data were analyzed using GraphPad Prism® software version 5.01 (GraphPad Software Inc., San Diego, CA, United States).

## Results

### HPLC Analysis

**FIGURE 1:**
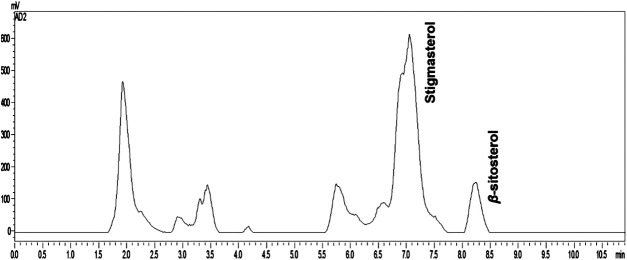
Chromatogram (HPLC-ELSD) of *Oceanapia magna* fraction.

In the chromatogram regarding the quantification of β-sitosterol and stigmasterol steroids, it was possible by high performance liquid chromatography–evaporative light-scattering detector (HPLC-ELSD). The chromatogram shows that the concentration of stigmasterol is greater than β-sitosterol and can be considered as chemical marker for this specie. Curiously, these compounds are isolated from plant species always together, however in this work it was possible to analyze and quantify the two separate constituents using C-18 column coupled to HPLC-ELSD (Figure 1; Table 1).

**TABLE 1:** Quantification of stigmasterol and *β*-sitosterol from Oceanapia magna extract.

	100.0 (µg)of extract	R.T.	R^2^
Stigmasterol	1.97	7.052	0.996
*β*-sitosterol	0.26	8.236	0.995

R.T., retention time; R^2^, correlation coefficient.

The calibration curve was based on peak area and using external standard in concentrations ranging from 0.0156 to 0.125 mg/ml, for both β-sitosterol (Sigma) and stigmasterol (Merck), both in triplicate. The limit of detection (LOD) and the limit of quantification (LOQ) for *β*-sitosterol were 0.135 and 0.408 μg, respectively, and for stigmasterol were 0.131 and 0.396 µg, respectively.

### Effect of OC-EtOH on Basal Tonus of Guinea Pig Ileum

**FIGURE 2:**
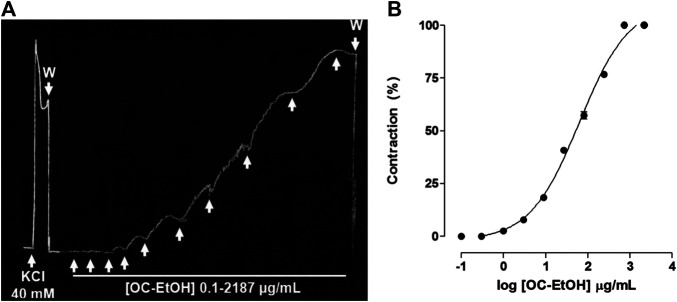
Representative record of OC-EtOH (0.1–2,187 μg/ml, n = 5) cumulative contractile effect on basal tonus of guinea pig ileum **(A)**. Cumulative concentration-response curves to OC-EtOH (● on basal tonus of guinea pig ileum (n = 5) **(B)**. The upward arrows represent the concentration of 0.1; 0.3; 1; 3; 9; 27; 81; 243; 729 and 2,187 μg/ml of OC-EtOH. W, wash.

OC-EtOH (0.1–2187 μg/ml, n = 5), cumulatively added on basal tonus, contracted guinea pig ileum in a concentration-dependent manner (EC_50_ = 48.6 ± 2.7 μg/ml) with a similar amplitude to previous contraction induced by KCl 40 mM (Figure 2A). Maximum contractile effect (E_max_) of the OC-EtOH was obtained with 729 μg/ml (Figure 2).

### Evaluation of Muscarinic Receptors Participation in Spasmogenic Effect of OC-EtOH

**FIGURE 3:**
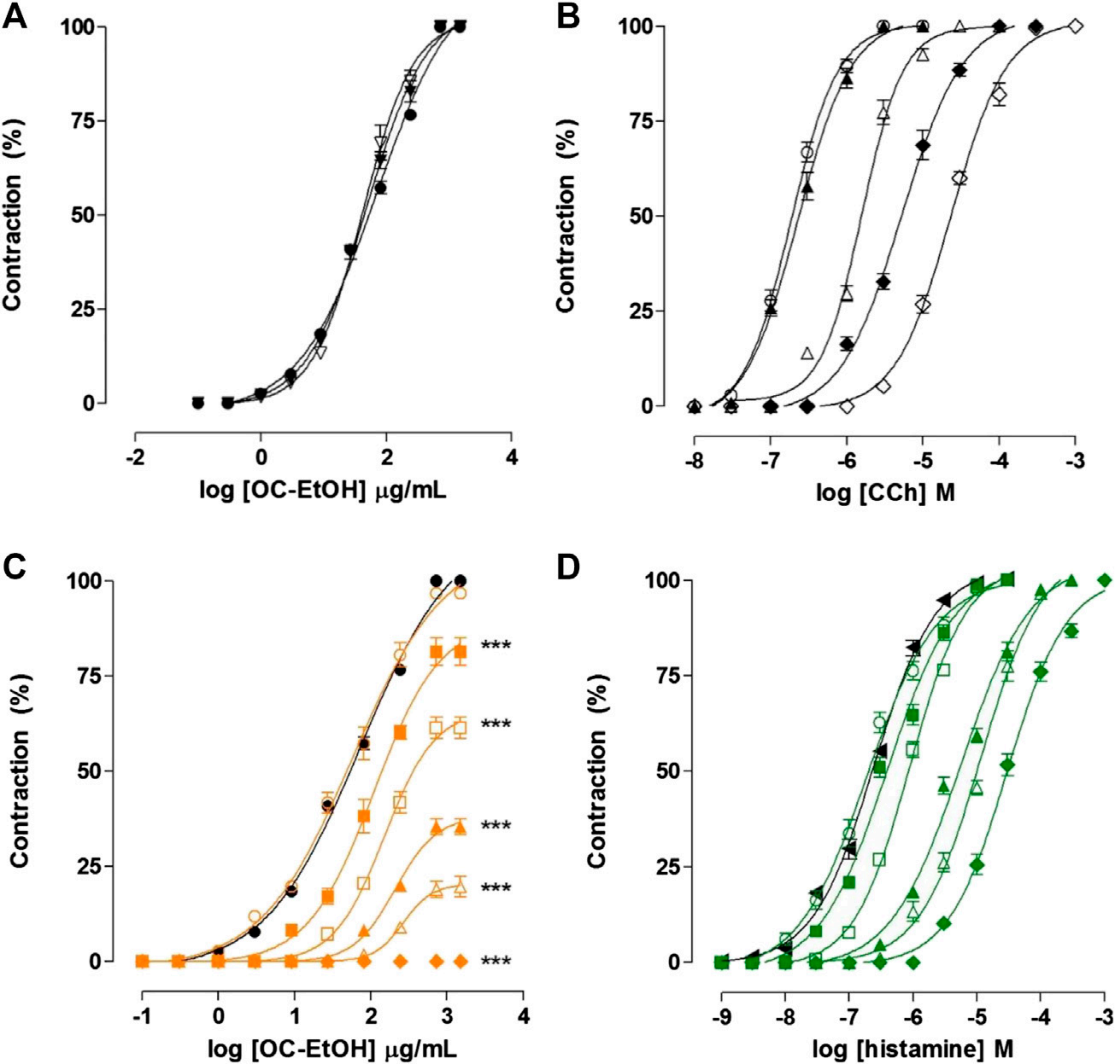
Cumulative concentration–response curves to OC-EtOH **(A,C)**, CCh **(B)** and histamine **(D)** on guinea pig ileum in both absence (○, ●, ◀, ●) and presence of atropine 10^–9^ (▲), 3 × 10^–9^ (△), 10^–8^ (◆, ▼) and 3 × 10^–8^ M (◇, ▽) or pyrilamine 10^–9^ (green unfiled circle, orange unfiled circle), 3 × 10^–9^ (green filed square, orange filed square), 10^–8^ (green unfiled square, orange unfiled square), 3 × 10^–8^ (green filed triangle, orange filed triangle), 10^–7^ (green unfiled triangle, orange unfiled triangle) and 3 × 10^–7^ M (green filed diamond, orange filed diamond) on guinea pig ileum. Symbols and vertical bars represent the mean and S.E.M., respectively (n = 5). CCh = Carbachol. One-way ANOVA followed by Bonferroni’s post-test: ***p < 0.001 (control vs. pyrilamine).

In presence of atropine (10^–8^ and 3 × 10^–8^ M), a non-selective muscarinic antagonist (positive control), OC-EtOH-induced cumulative contraction curve (EC_50_ = 48.6 ± 2.7 μg/ml, n = 5) was not shifted (EC_50_ = 43.3 ± 2.6 and 41.4 ± 3.8 μg/ml, respectively, n = 5) and E_max_ was not modified (Figure 3A).

Differently, in presence of atropine (positive control), CCh-induced cumulative contraction curve (10^−8^–3 × 10^–5^ M, n = 5) was shifted to the right in a parallel manner, without E_max_ reduction. EC_50_ values for CCh were 1.9 ± 0.1 × 10^–7^ M in the absence and 2.4 ± 0.2 × 10^–7^; 1.5 ± 0.07 × 10^–6^; 5.3 ± 0.4 × 10^–6^ and 2.3 ± 0.04 × 10^–5^ M in the presence of 10^–9^; 3 × 10^–9^; 10^–8^ and 3 × 10^–8^ M of atropine, respectively (Figure 3B).

### Evaluation of Histamine Receptors Participation in Spasmogenic Effect of OC-EtOH

In presence of pyrilamine, a H_1_ histamine receptors antagonist (positive control), the OC-EtOH-induced cumulative contraction curve (10^–9^–3 × 10^–7^ M) was inhibited and shifted to the right in a non-parallel manner and presented E_max_ reduction of 100% (control) to 96.7 ± 1.1; 81.4 ± 3.6; 61.3 ± 2.8; 35.5 ± 2.1; 19.7 ± 2.7 and 0%, respectively (n = 5). The EC_50_ values of OC-EtOH were altered from 48.6 ± 2.7 μg/ml (control) to 41.3 ± 3.2; 88.6 ± 10; 131.2 ± 11 and 255.2 ± 25.7 μg/ml using the following pyrilamine concentrations 10^–9^, 3 × 10^–9^, 10^–8^, 3 × 10^–8^, 10^–7^ and 3 × 10^–7^ M, respectively (Figure 3C).

In the same way, in pyrilamine presence (positive control), histamine-induced cumulative contraction curve (10^−9^–10^–3^ M) was shifted to the right in a parallel manner without E_max_ reduction (n = 5). The EC_50_ values to histamine was 2.3 ± 0.2 × 10^–7^ M in absence and 2.5 ± 0.3 × 10^–7^; 3.9 ± 0.3 × 10^–7^; 8.3 ± 0.3 × 10^–7^; 5.0 ± 0.2 × 10^–6^; 9.5 ± 0.2 × 10^–6^ and 3.1 ± 0.3 × 10^–5^ M in presence of 10^–9^; 3 × 10^–9^; 10^–8^; 3 × 10^–8^; 10^–7^ and 3 × 10^–7^ M of pyrilamine, respectively (Figure 3D).

### Evaluation of Ca_V_ Participation in OC-EtOH Spasmogenic Effect

**FIGURE 4:**
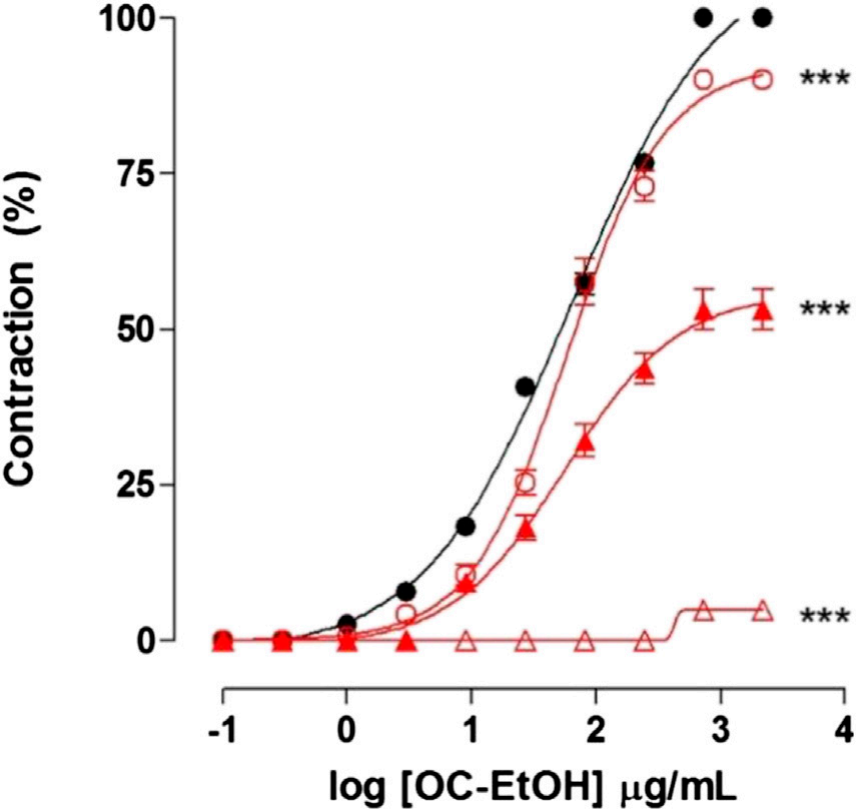
Cumulative concentration–response curves to OC-EtOH in absence (●) and presence of verapamil 10^–7^ (**◯**), 3 × 10^–7^ (▲) and 10^–6^ (**△**) on guinea pig ileum. Symbols and vertical bars represent the mean and S.E.M., respectively (n = 5). One-way ANOVA followed by Bonferroni's post-test: ***p < 0.001 (control vs. verapamil).

OC-EtOH-induced cumulative contraction curve (EC_50_ = 48.6 ± 2.7 μg/ml, n = 5) was inhibited in the presence of 10^–7^, 3 × 10^–7^ and 10^–6^ M of verapamil, a Ca_V_ blocker, with abolition of OC-EtOH contractile effect (Figure 4).

According to EC_50_ values of OC-EtOH, no differences between control curve (EC_50_ = 48.6 ± 2.7 μg/ml, n = 5) and those obtained in presence of 10^–7^ and 3 × 10^–7^ M of verapamil (EC_50_ = 55.9 ± 5.3 and 51.8 ± 3.7 μg/ml, respectively, n = 5) were observed. It was not possible to calculate the extract EC_50_ when the organ was incubated with 10^–6^ M of verapamil. E_max_ values were altered in the presence of 10^–7^; 3 × 10^–7^ and 10^–6^ M of verapamil from 100% (control) to 90.0 ± 1.2; 53.2 ± 3.2 and 4.9 ± 1.3%, respectively (Figure 4).

### Effect of OC-EtOH on KCl-, Histamine- or Carbachol-Induced Tonic Contractions

**FIGURE 5:**
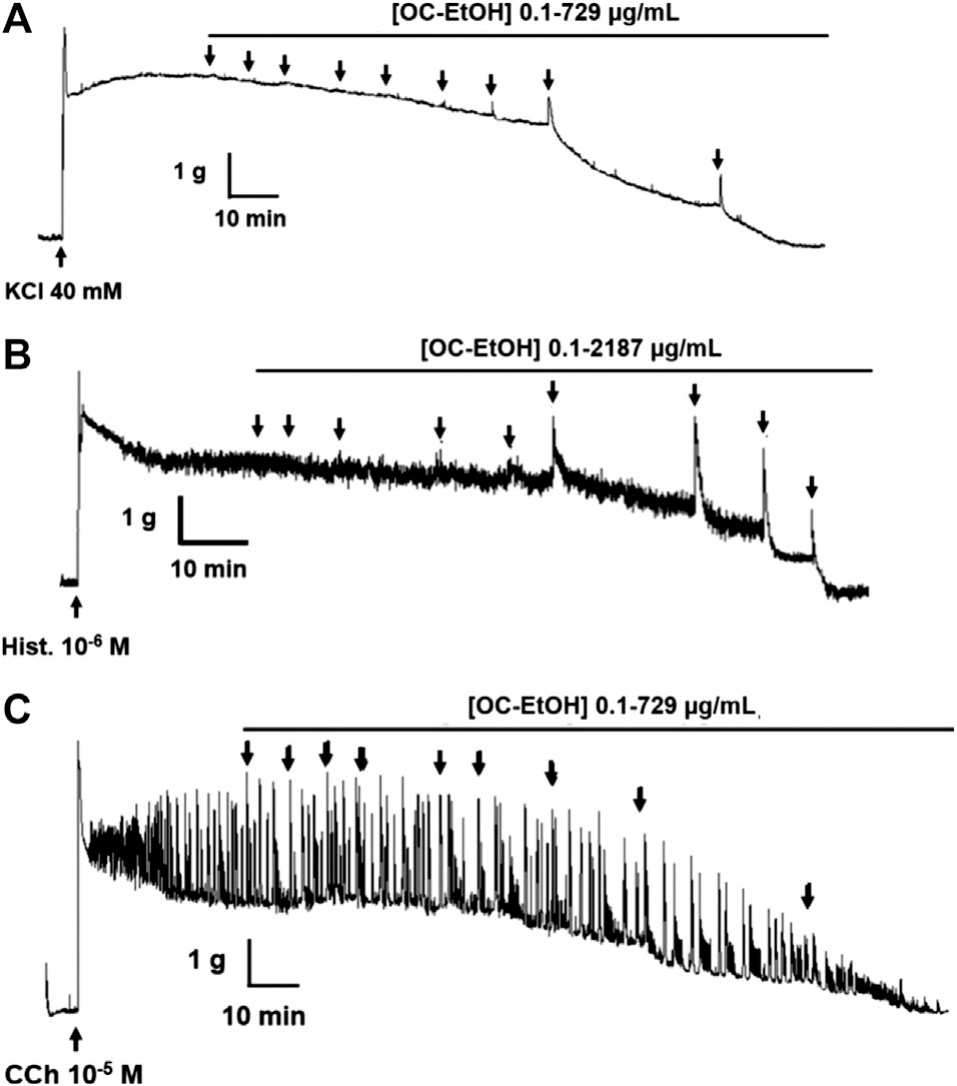
Representative records of OC-EtOH relaxant effect on guinea pig ileum pre-contracted by KCl 40 mM **(A)**, histamine 10^–6^ **(B)** or CCh 10^–5^ M **(C)** (n = 5).

**FIGURE 6:**
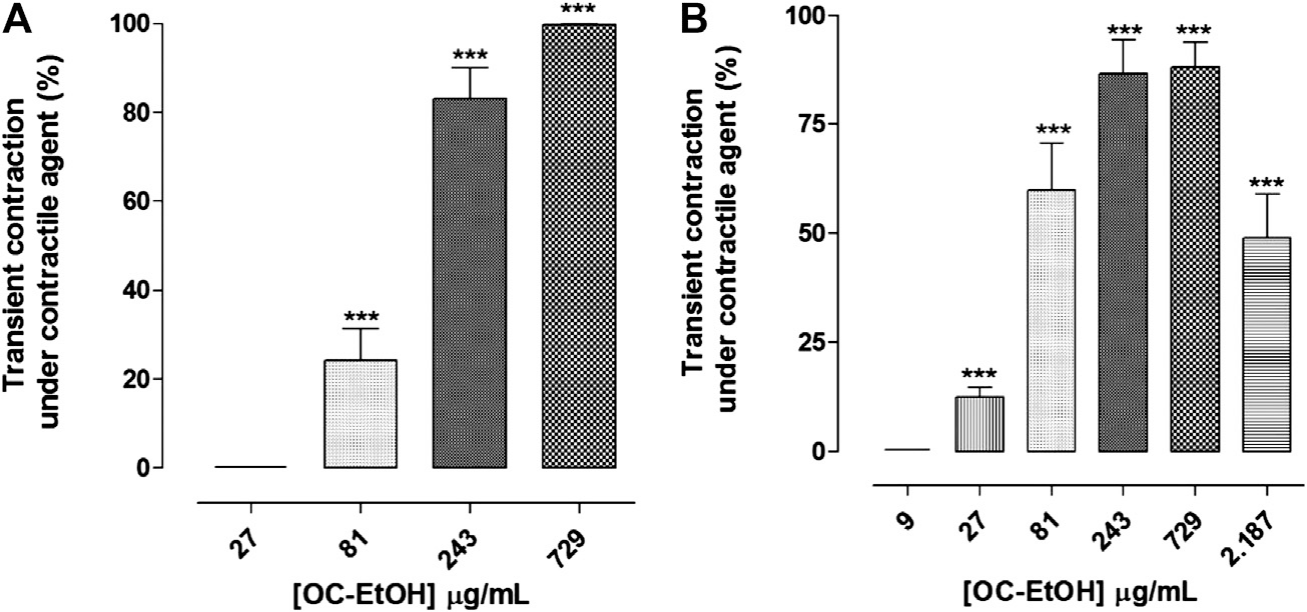
OC-EtOH contractile effect under KCl-**(A)** and histamine-**(B)** induced tonic contractions on guinea pig ileum. Vertical bars represent the means and S.E.M., respectively. One-way ANOVA followed by Bonferroni’s post-test, significant differences are indicated by ***p < 0.001 (n = 5).

OC-EtOH (0.1–2187 μg/ml, n = 5) induced a transient contractile effect (spasmogenic) followed by a relaxation (spasmolytic) in a concentration-dependent manner when guinea pig ileum was pre-contracted both with KCl 40 mM or histamine 10^–6^ M (Figure 5). Transient contraction induced by OC-EtOH at 27, 81, 243 and 729 μg/ml were 0.0; 24.2 ± 7.1; 83.1 ± 6.9 and 99.8 ± 0.1%, respectively, compared to tonic component induced by KCl and 12.4 ± 2.2; 59.8 ± 10.7; 86.5 ± 7.9 and 88.1 ± 5.8%, respectively, compared to histamine (Figure 6). OC-EtOH spasmogenic potency was around twice more potent when the contraction was induced by histamine (EC_50_ = 73.5 ± 6.9 μg/ml) than by KCl (EC_50_ = 176.6 ± 32.6 μg/ml) (Figure 6).

**FIGURE 7:**
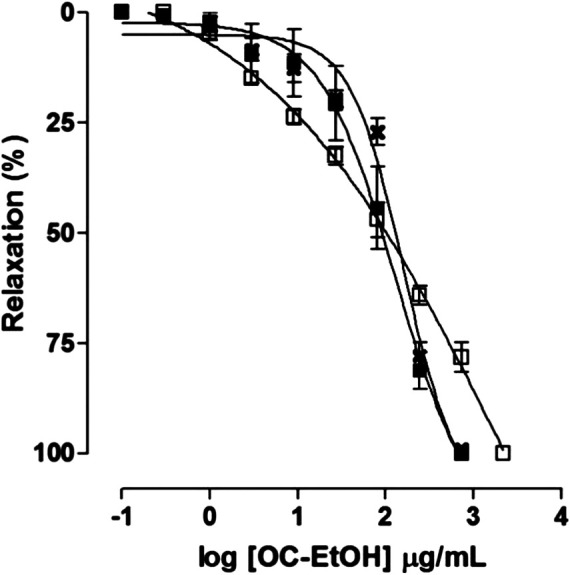
Effect of OC-EtOH on guinea pig ileum pre-contracted with KCl 40 mM (✖), histamine 10^–6^ M (□) or CCh 10^–5^ M (■) (n = 5). Symbols and vertical bars represent the mean and S.E.M., respectively.

Differently, on tonic contraction induced by CCh 10^–5^ M, OC-EtOH (0.1–729 μg/ml, n = 5) showed only a relaxant effect profile (Figure 5). About OC-EtOH spasmolytic potency, it was equipotent to relax guinea pig ileum pre-contracted either by KCl (EC_50_ = 103.9 ± 8.6 μg/ml), histamine (EC_50_ = 90.1 ± 9.2 μg/ml) or CCh (EC_50_ = 97.1 ± 17.4 μg/ml). The OC-EtOH spasmolytic E_max_ (100%) was obtained at 729 μg/ml when guinea pig ileum was contracted by KCl or CCh, and at 2187 μg/ml when the organ was contracted by histamine (Figure 7).

### Effect of OC-EtOH on KCl-Induced Tonic Contraction in the Presence of Pyrilamine

**FIGURE 8:**
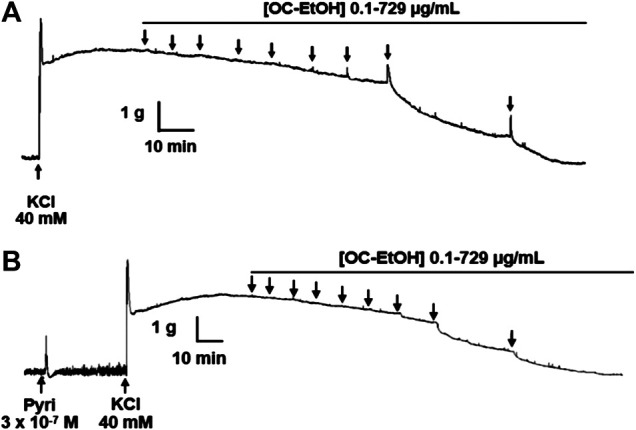
Representative records of OC-EtOH relaxant effect on guinea pig ileum pre-contracted with KCl 40 mM in absence **(A)** and presence **(B)** of pyrilamine (n = 5). Pyri, pyrilamine.

**FIGURE 9:**
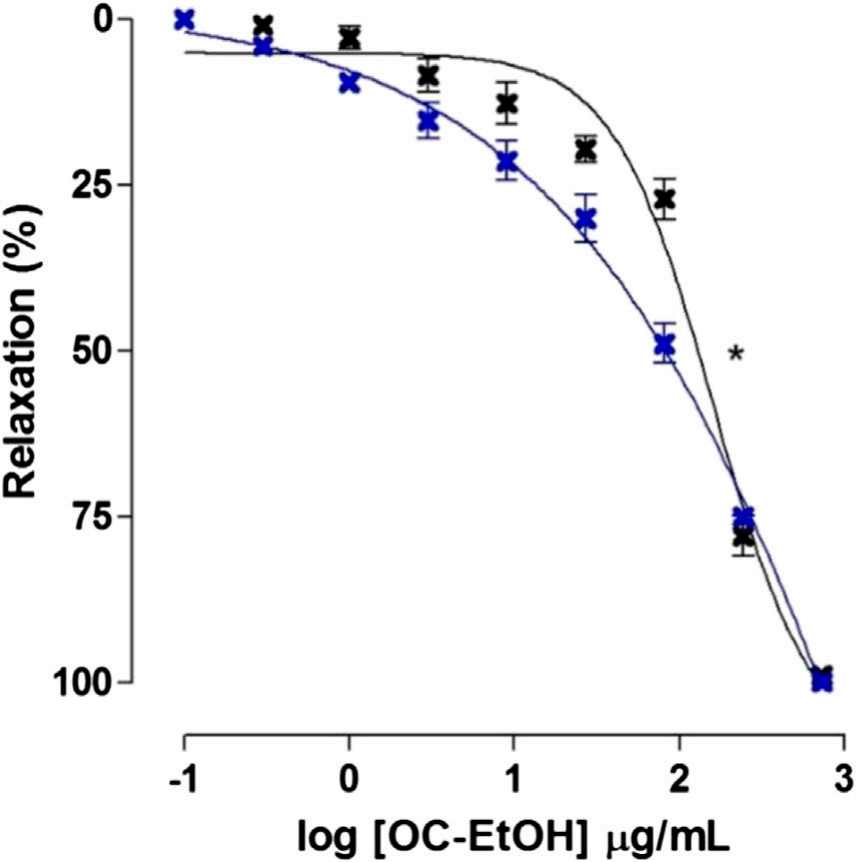
Relaxant effect of OC-EtOH on guinea pig ileum pre-contracted with KCl 40 mM in absence (black cross) and presence (blue cross) of pyrilamine. Symbols and vertical bars represent the mean and S.E.M., respectively (n = 5). One-way ANOVA followed by Bonferroni’s post-test: *p < 0.05 (KCl vs. pyrilamine + KCl).

In the presence of pyrilamine (3 × 10^–7^ M), the transient contractile effect of OC-EtOH on KCl-induced tonic contraction was abolished (Figure 8). Interestingly, in presence of pyrilamine, OC-EtOH spasmolytic effect was potentiated (Figure 9).

According to EC_50_ values, OC-EtOH was about 2-fold more potent in relaxing KCl-induced tonic contraction in the presence of pyrilamine 3 × 10^–7^ M (EC_50_ = 62.9 ± 9.9 μg/ml, n = 5) than in its absence (EC_50_ = 103.9 ± 8.6 μg/ml, n = 5).

### Effect of OC-EtOH on CaCl_2_-Induced Contractions in Depolarizing Medium Nominally Without Ca^2+^

**FIGURE 10:**
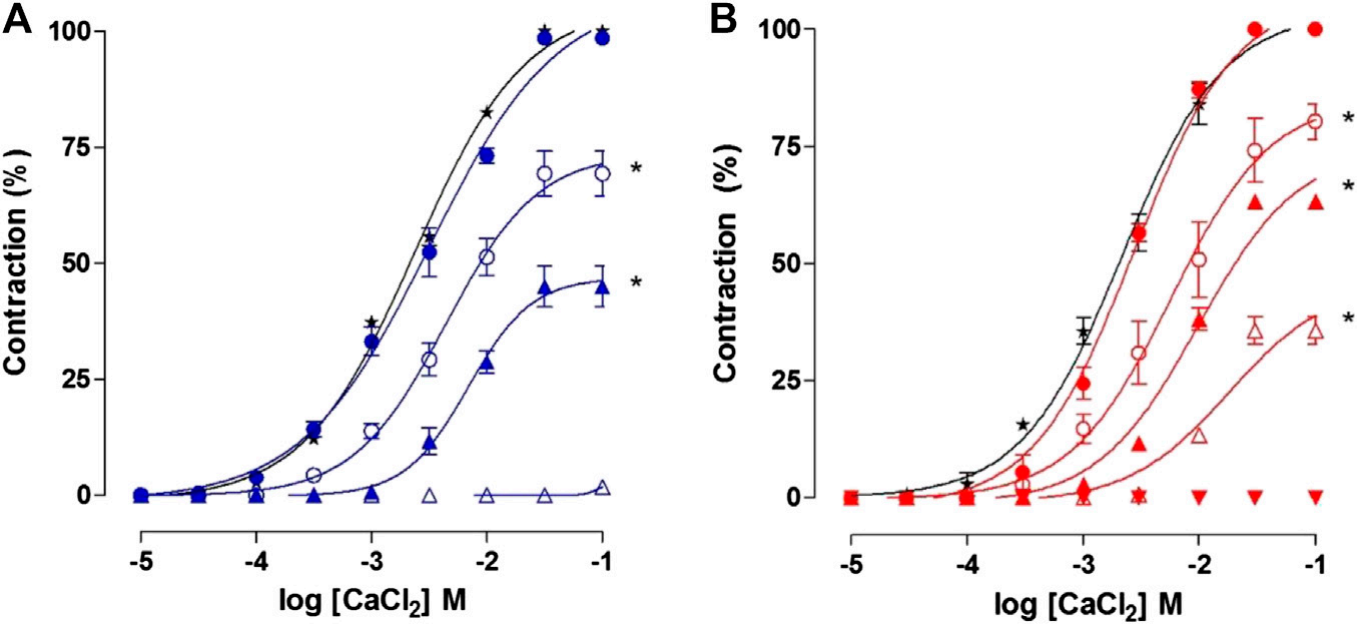
Cumulative concentration–response curves to CaCl_2_ in depolarizing medium nominally without Ca^2+^ in absence (**★**) and presence of 27 (blue filed circle), 81 (blue unfiled circle), 243 (blue filed triangle) and 729 μg/ml (blue unfiled triangle) of OC-EtOH **(A)** and in presence of 3 × 10^–8^ (red filed circle), 10^–7^ (red unfiled circle), 3 × 10^–7^ (red unfiled triangle10^–6^ (red filed triangle) and 3 × 10^–6^ M (red inverted filed triangle) of verapamil **(B)** on guinea pig ileum. Symbols and vertical bars represent the mean and S.E.M., respectively (n = 5). One-way ANOVA followed by Bonferroni's post-test: *p < 0.05 (control *vs.* verapamil or OC-EtOH).

OC-EtOH (27, 81, 243 and 729 μg/ml, n = 5) inhibited the contractions induced by cumulative increase in extracellular concentration of CaCl_2_ in depolarizing medium nominally without Ca^2+^ (n = 5). CaCl_2_ cumulative concentration-response curves were shifted to the right in a non-parallel manner and with E_max_ reduction from 100% (control) to 98.5 ± 0.9; 69.3 ± 4.8; 45.0 ± 4.4 and 1.8 ± 0.5%, respectively. EC_50_ values of the CaCl_2_ were altered from 2.1 ± 0.1 × 10^–3^ M (control) to 2.5 ± 0.3; 3.9 ± 0.7 and 6.8 ± 1.4 × 10^–3^ M, respectively. The EC_50_ could not be determined when the organ was incubated with 729 μg/ml of the extract t (Figure 10A).

Similarly, verapamil, a Ca_V_ blocker (positive control; 3 × 10^–8^–3 × 10^–6^ M) inhibited CaCl_2_ cumulative concentration-response curves (10^−5^–10^–1^ M). The curves were rightwards shifted, in a non-parallel manner, presenting E_max_ reduction from 100% (control) to 80.2 ± 3.7; 63.2 ± 0.9; 35.6 ± 2.9 and 0%, respectively. EC_50_ values of the CaCl_2_ were altered from 2.1 ± 0.3 × 10^–3^ M (control) to 2.4 ± 0.2; 3.5 ± 0.4; 7.4 ± 0.03 × 10^–3^ and 1.2 ± 0.01 × 10^–2^ M; respectively (Figure 10B).

### Effect of OC-EtOH on S-(-)-Bay K8644-Induced Tonic Contractions

**FIGURE 11:**
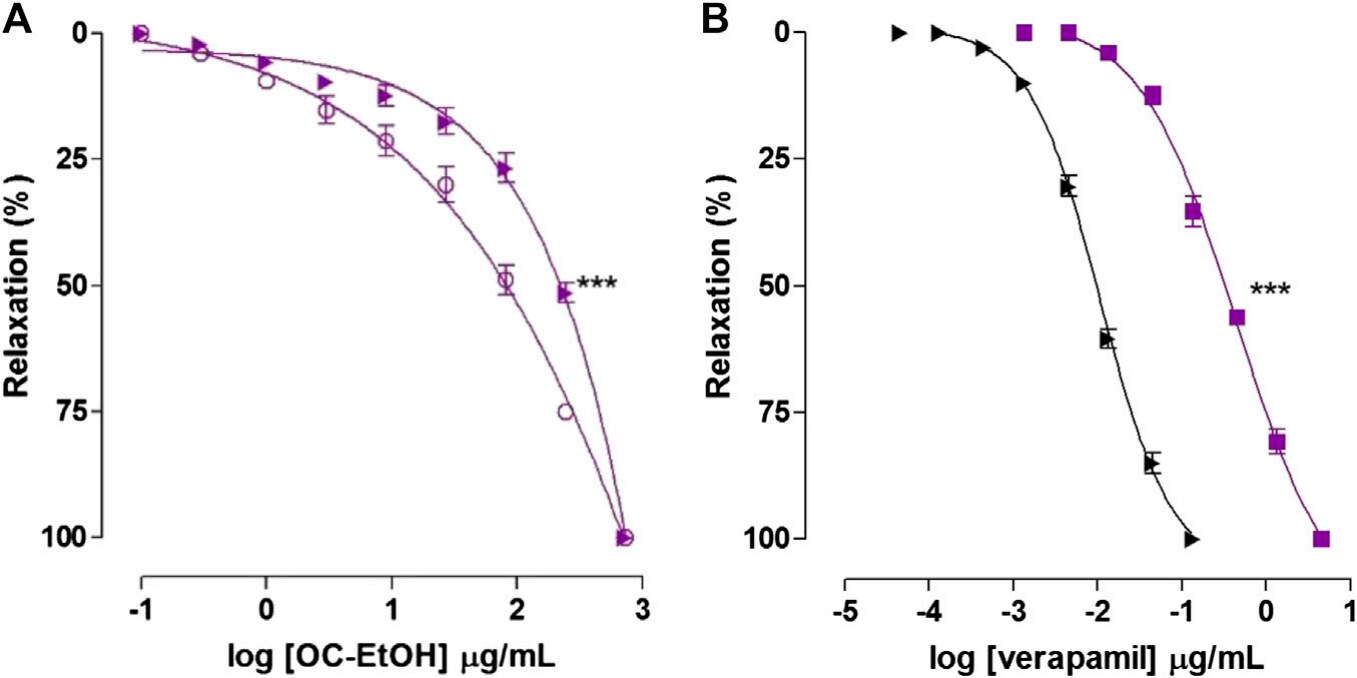
Relaxant effect of OC-EtOH **(A)** on guinea pig ileum pre-contracted with KCl 40 mM (violet unfiled circle) and S-(-)-Bay K8644 3 × 10^–7^ M (violet right-pointing triangle) in pyrilamine presence and relaxant effect of verapamil **(B)** on guinea pig ileum pre-contracted with KCl 40 mM (black right-pointing triangle) and S-(-)-Bay K8644 3 × 10^–7^ M (violet filed square) in pyrilamine presence. Symbols and vertical bars represent the mean and S.E.M., respectively (n = 5). One-way ANOVA followed by Bonferroni's post-test: ***p < 0.001 (S-(-)-Bay K8644 vs. KCl).

OC-EtOH relaxed guinea pig ileum pre-contracted with S-(-)-Bay K8644 3 × 10^–7^ M, a Ca_V_ agonist, in pyrilamine presence (EC_50_ = 166.9 ± 17.8 μg/ml, n = 5) and the relaxant potency of the extract was reduced by 2.6 times when compared to pre-contracted ileum with KCl 40 mM (EC_50_ = 62.9 ± 9.9 μg/ml n = 5) (Figure 11A).

As positive control, verapamil relaxed guinea pig ileum pre-contracted with KCl 40 mM (EC_50_ = 0.0094 ± 0.0006 μg/ml) and S-(-)-Bay K8644 3 × 10^–7^ M (EC_50_ = 0.30 ± 0.02 μg/ml, n = 5), in medium partially depolarized with KCl 15 mM (Figure 11B).

### Behavioral Pharmacological Screening and Evaluation of Acute Toxicity of OC-EtOH in Mice

After OC-EtOH oral administration (2,000 mg/kg), no behavioral changes were observed in female mice (n = 6) in the evaluated experimental conditions during 4 h of observation. Weight assessment were performed before the treatment, 7 and 14 days after the treatment and no changes in animals’ weight were observed in both negative control animals (33.5 ± 0.3, 33.7 ± 0.8 and 34.8 ± 0.6 g) and in the animals treated with OC-EtOH (29.0 ± 0.5, 30.0 ± 0.7 and 30.7 ± 0.6 g)., respectively. Interestingly, there were no death events during 14 days of observation.

### Effect of OC-EtOH on Upper Gastrointestinal Transit in Mice

**FIGURE 12:**
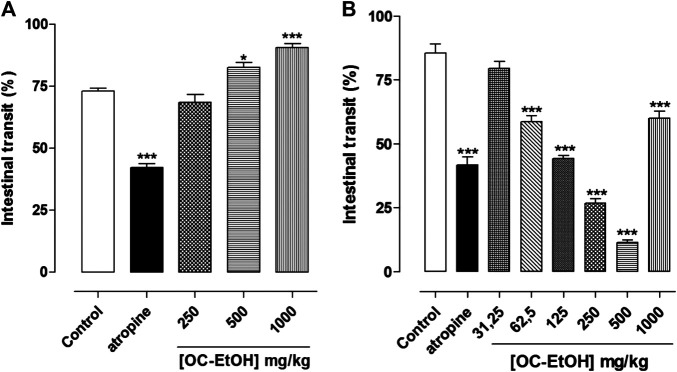
Effect of OC-EtOH on normal **(A)** and castor-oil-induced **(B)** upper gastrointestinal transit in mice (n = 6). Columns and vertical bars represent the mean and S.E.M., respectively. One-way ANOVA followed by Bonferroni’s post-test: *p < 0.05 and ***p < 0.001 (saline vs. atropine/OC-EtOH).

OC-EtOH (250, 500 and 1,000 mg/kg, p.o.) increased dose dependently the intestinal transit traveled by the activated charcoal (marker) (72.9 ± 1.2%) to 82.6 ± 1.8 and 90.5 ± 1.5%, respectively, showing an ED_50_ of 477.6 ± 14.8 mg/ml (n = 6). The distance traveled by the marker in the intestine of control animals (72.9 ± 1.2% of intestine total length) was decreased to 42.2 ± 1.4% when the animals were treated with atropine (2 mg/kg, p.o.) (Figure 12A).

In contrast, OC-EtOH (62.5; 125, 250, 500 and 1,000 mg/kg) inhibited the castor oil-induced intestinal transit, in a dose-dependent manner, reducing the distance in 41.3 ± 2.3; 55.6 ± 1.1; 73.2 ± 1.8; 88.5 ± 0.9 and 40.0 ± 2.8% (n = 6), respectively, in comparison to the negative control (0.9% saline + Cremophor^®^). It was also observed that atropine (2 mg/kg) inhibited 58.2 ± 3.1% of the distance traveled by the marker in the castor oil-induced intestinal transit as compared to the negative control (Figure 12B). OC-EtOH showed ED_50_ of 93.30 ± 7.2 mg/kg and the E_max_ (88.5 ± 0.9%) was observed at 500 mg/kg (n = 6).

### Effect of OC-EtOH on Castor Oil-Induced Diarrhea in Mice

**FIGURE 13:**
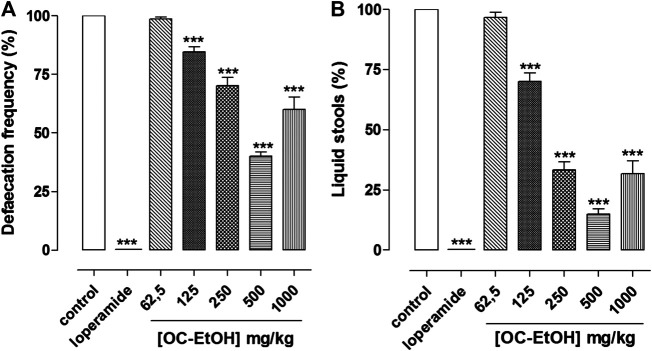
Antidiarrheal effect of OC-EtOH on castor-oil-induced diarrhea in mice (n = 6). Percentage of total stools number **(A)** and percentage of liquid stools **(B)**. Columns and vertical bars represent mean and S.E.M., respectively. One-way ANOVA followed by Bonferroni’s post-test: ***p < 0.001 (saline vs. loperamide/OC-EtOH).

OC-EtOH (125, 250, 500 and 1,000 mg/kg, p.o., n = 6) inhibited castor oil-induced diarrhea equipotently and in a dose-dependent manner in terms of defecation frequency (15.4 ± 2.2; 29.8 ± 3.6; 59.8 ± 1.6 and 40.0 ± 5.3%, respectively) and number of liquid stools (30.0 ± 3.6; 66.7 ± 3.3; 85.0 ± 2.2 and 68.3 ± 5.4%) when compared to the negative control. It was also observed that loperamide (positive control, 10 mg/kg) inhibited 100% of castor oil-induced diarrhea (Figure 13). OC-EtOH showed ED_50_ and E_max_ 2-3-fold more potent in inhibiting liquid stool (ED_50_ = 189.1 ± 13.6 mg/ml, E_max_ = 15.0 ± 2.2%) compared to defecation frequency (ED_50_ = 387.7 ± 21.6 mg/ml, E_max_ = 40.2 ± 1.6%).

## Discussion

In this work, the effects of ethanolic extract obtained from *Oceanapia magna*. sponge (OC-EtOH) were investigated on rodents’ intestinal smooth muscle. It was demonstrated, for the first time, that OC-EtOH has dual effect on gastrointestinal tract, presenting spasmogenic action in guinea pig ileum, through histamine receptors (H_1_ subtype) activation, and spasmolytic effect by blocking the influx of Ca^2+^ through voltage-gated calcium channels (Ca_V_1). Therefore, *Oceanapia magna*. sponge demonstrated to be a potential source for medicinal metabolites that could be used for intestinal diseases treatment, such as constipation and/or diarrhea.

Initially, the effect of OC-EtOH was assessed on guinea pig ileum basal tonus in order to evaluate its possible activity on intestinal smooth muscle. Thus, the extract promoted intestinal contraction in a concentration-dependent manner (Figure 2), showing the spasmogenic effect of OC-EtOH.

In view of these results, an investigation of the mechanism that leads the tested product to promote a contraction was carried out. One of the possible spasmogenic mechanisms could involve the activation of M3 and/or H1 receptors. These receptors are characterized as receptors coupled to G_q/11_ protein-PLCβ effector system, resulting in an increase of intracellular Ca^2+^ concentration, leading to muscle contraction (Ehlert et al., 1999; Berridge et al., 2003). To evaluate the muscarinic receptors participation in spasmogenic effect, we used the standard drug atropine, a nonselective muscarinic receptors antagonist (Bashir et al., 2006). According to the results, it was observed that atropine, in different concentrations, did not inhibit OC-EtOH-induced cumulative contraction curves. Thus, muscarinic receptors activation by OC-EtOH was discarded in its contractile effect (Figure 3).

H_1_ histamine receptors are distributed in a variety of tissues, including airways, genitourinary, cardiovascular and gastrointestinal smooth muscle (Hill, 1990). To evaluate the hypothesis that OC-EtOH could act on H_1_ receptors to produce its contractile effect, pyrilamine (H_1_ receptor antagonist) was used as standard drug (Ghayur and Gilani, 2005). It was found that OC-EtOH-induced cumulative contraction curves in pyrilamine presence were shifted to right with reduction on both potency and efficacy. According to appearance of curves, it can be said that in OC-EtOH there are components that appear to act via H_1_ receptors, however there are other components that act in other ways (Figure 3).

An increase of [Ca^2+^]_c_ in the smooth muscle is the main cause for contraction production (Hill-Eubanks et al., 2011). Ca_V_ are an essential element in initialization and/or maintenance of smooth muscle contraction. So, we decided to evaluate if Ca_V_ could be involved in the OC-EtOH spasmogenic mechanism of action. Verapamil, a Ca_V_ blocker, was used as a standard drug (Staneva-Stoytcheva and Venkova, 1992). OC-EtOH was added in a cumulative manner in the presence of different concentrations of verapamil and the curves were shifted to the right, with a reduction in both potency and efficacy of OC-EtOH (Figure 4). It can be suggested that the secondary metabolites found in OC-EtOH are activating Ca_V_ to promote its spasmogenic effect on guinea pig ileum.

Guinea pig ileum has a biphasic contraction (phasic and tonic) and, particularly, this organ depends on extracellular Ca^2+^ and the influence of this ion is higher on the tonic response (Triggle et al., 1979; Honda et al., 1996). Therefore, we decided to also evaluate the effect of OC-EtOH on tonic component of the contraction induced by different contractile agents such as KCl, histamine and CCh on guinea pig ileum.

The OC-EtOH presented a dual profile on tonic contraction induced by KCl and histamine: in the first moment, the extract induced a transient contraction followed by a slow relaxation, both in a concentration-dependent manner (Figure 5). No significant difference between the values for the spasmolytic extract potency was observed (Figure 7) and this indicates that OC-EtOH has secondary metabolites that act in a common pathway to modulate the intestinal smooth muscle motility. OC-EtOH showed a higher spasmogenic potency when compared to its spasmolytic effectiveness. These findings suggest, for the first time, that OC-EtOH, a marine natural product, presents dual effect (spasmogenic and spasmolytic) on guinea pig intestinal smooth muscle.

As showed until now, OC-EtOH possibly presents secondary metabolites with dual effect on intestinal smooth muscle. So, it was decided to proceed with the investigation of the mechanisms underlying the OC-EtOH spasmolytic activity.

To verify if the OC-EtOH spasmogenic component was interfering in the relaxant action, the experimental protocols were carried out in the presence of pyrilamine to avoid the OC-EtOH spasmogenic activity. The OC-EtOH-induced contraction was abolished in the presence of the H_1_-antagonist, and the OC-EtOH relaxation effect on tonic contraction induced by KCl was about 2-fold more potent when compared to relaxation in antagonist absence (Figures 8,9). Therefore, this evidence suggests that the transient contractile effect promoted by OC-EtOH is due to a positive modulation of H_1_ receptors on intestinal smooth muscle.

Smooth muscle is described as a biphasic organ, presenting two phases of contraction. In the first one, the contraction is rapid and transient. The second phase is characterized by a tonic contraction that is maintained for a while (Horie et al., 2005). Both phases are dependent on extracellular Ca^2+^, as the whole contraction is inhibited by blocking Ca_V_, which means that this is one of the main pathways involved in this entry (Rembold, 1996). Since OC-EtOH inhibited the tonic contractions induced by three distinct contractile agents in a concentration-dependent manner, presenting no difference among the pharmacological potencies (Figure 7), we theorized that OC-EtOH might be acting on Ca_V_, by blocking Ca^2+^ influx once both coupling mechanisms, mixed (histamine and CCh) and electromechanical (KCl), lead to tonic contractions almost exclusively by Ca^2+^ influx through Ca_V_ (Bolton, 1979; Rembold, 1996; Bolton, 2006).

Verapamil was used as positive control since it is a standard drug that blocks Ca_V_. In presence of OC-EtOH, cumulative concentration-response curves to CaCl_2_ were shifted to right, in a non-parallel manner, and reduced E_max_ (Figure 10). The results obtained were similar to the positive control. These findings support the hypothesis that OC-EtOH could inhibit Ca^2+^ influx through Ca_V_ to produce its spasmolytic effect.

To reinforce this hypothesis, OC-EtOH was assessed in relaxing the guinea pig ileum pre-contracted by S-(-)-Bay K8644 (Conte-Camerino et al., 1987), a Ca_V_1 agonist (Ferrante et al., 1989). The experimental protocol was carried out in pyrilamine presence, in order to remove the spasmogenic effect of the extract, and verapamil was used as positive control. OC-EtOH relaxed the organ pre-contracted with S-(-)-Bay K8644 in a concentration-dependent manner and it was 2.6 times less potent when the organ was pre-contracted with KCl (Figure 11). So, surely, OC-EtOH blocked the Ca^2+^ channels to induce its spasmolytic effect on guinea pig ileum, and Ca_V_1 is the channel subtype involved, but other targets are not discarded in the participation of OC-EtOH dual effect on intestinal smooth muscle.

Following the *in vitro* investigation, female mice received acute treatment of OC-EtOH 2,000 mg/kg (n = 6, p.o.) and animals were observed for 4 h. No behavioral changes were observed, discarding toxic effect on central and autonomic nervous systems. There was no death of animals during 14 days of observation and no interference in ponderal evolution of animals. This assay was divided into two groups with three animals each. So, in the dosing regimen used (2,000 mg/kg), OC-EtOH showed no toxic effect, and, according to 423/2001 Organization for Economic Cooperation and Development (OEDC) guideline, this result indicates a low acute toxicity and an estimate of LD_50_ greater than or equal to 5,000 mg/kg. This result shows that OC-EtOH can be considered safe for the following *in vivo* experimental protocols.

Since OC-EtOH presented spasmogenic and spasmolytic activity in intestinal smooth muscle and diarrhea and constipation can be caused, among other factors, due to changes in intestinal motility (Field and Semrad, 1993), we decided to investigate if OC-EtOH alters the intestinal motility in mice.

Interestingly, OC-EtOH did not inhibit normal upper gastrointestinal transit in mice, but an increase of the intestinal distance traveled by the marker was observed when animals were treated with doses of 500 and 1,000 mg/kg when compared to control group. Because at the dose of 500 mg/kg the observed effect was not 100%, the 1,000 mg/kg dose was used to make sure that the previous dose would be the maximum or not. The fact that the dose of 1,000 mg/ml gave a higher value can be explained by the desensitization of the organ or system, showing that the dose of 500 mg/kg is the one that produces the maximum effect. Given these results and corroborating data found *in vitro* assays, it may be suggested the OC-EtOH promotes a laxative effect under physiological conditions. Different results were observed in castor-oil-induced intestinal transit, since, in this biological context, OC-EtOH inhibited dose-dependently intestinal motility in mice (Figure 12). This fact may be directly related to an antidiarrheal effect and this might occur by intestinal motility inhibition, in pathological conditions, what could represent a relevant therapeutic effect. This effect can help to reduce the number of evacuations, increase water absorption due to slow passage of intestinal contents, and increase stool viscosity (Gurgel et al., 2007).

It is reported that castor oil inhibits Na^+^/K^+^-ATPase, reducing the absorption of normal liquid (Izzo et al., 1999), in addition to promoting the activation of adenylyl cyclase, stimulating the production of cAMP (Gaginella and Bass, 1978). Ricinoleic acid is also responsible for producing irritating and inflammation of the intestinal mucosa, leading to the release of several inflammatory mediators, such as prostaglandins, nitric oxide (NO), platelet activation factor (PAF) and tachykinins (Awouters et al., 1978). In addition, the ricinoleic acid is an agonist of prostaglandin E_2_ (PGE_2_) receptors, activating EP_3_ and EP_4_, and the activation of the former in intestinal smooth muscle is responsible for promoting its diarrheal effect. OC-EtOH also inhibited castor oil-induced diarrhea in a concentration-dependent manner by inhibiting both defecation frequency and liquid feces. The extract was twice more potent and effective in inhibiting watery stools compared with total defecation frequency. This fact is also quite interesting, as it may suggest that OC-EtOH would be interfering in one of the aforementioned ricinoleic acid-activated pathways, acting on liquid stools formation without preventing normal feces formation, which could cause constipation (Figure 13).

In conclusion, OC-EtOH showed to be a safe marine product since it was assessed by an animal model of toxicity. For the first time, a biological activity was assigned for *Oceanapia magna* and it was observed that OC-EtOH presented dual effect (spasmogenic due to histamine receptors activation, and spasmolytic by Ca^2+^ influx through Ca_V_ blockade). Further studies are needed to verify other possible targets for the mechanism of action of OC-EtOH. Its potential benefits by acting in a more balanced way than compounds with a single target and, consequently, would show a satisfactory pharmacological combination effect and/or reduced side effects. Thus, *Oceanapia magna* sponge arises as an oceanic natural product with potential medical use to intestinal diseases, such as diarrhea.

## Conflicts of Interest

The authors declare that the research was conducted in the absence of any commercial or financial relationships that could be construed as a potential conflict of interest.

## Data Availability Statement

The raw data supporting the conclusions of this article will be made available by the authors, without undue reservation.

## Ethics Statement

The animal study was reviewed and approved by Ethics Committee on Animal Use (CEUA)/UFPB, certificate no. 146/2015.

## Author Contributions

JP, IF, FO, SF, and GM were involved in experimental work and preparation of the manuscript. BVS, UP, and TS contributed to obtaining and standardizing the OC-EtOH. BAS and FC elaborated the experimental design and all aspects of this project.

## Funding

The authors thank to Coordenação de Aperfeiçoamento de Pessoal de Nível Superior (CAPES) and to Conselho Nacional de Desenvolvimento Científico e Tecnológico (CNPq) for financial support and UFPB and PPgPNSB for experimental support.
